# The Impact of Climate-Related Natural Disasters and Extreme Weather Events on Plastic and Reconstructive Surgery: A Scoping Review

**DOI:** 10.7759/cureus.100499

**Published:** 2025-12-31

**Authors:** Hannah D Shi, Alex J Dong, Seungil Lee, LaYow C Yu, Carol Mita, Colby Hyland, Justin Broyles

**Affiliations:** 1 Plastic and Reconstructive Surgery, Harvard Medical School, Boston, USA; 2 Countway Library of Medicine, Harvard Medical School, Boston, USA; 3 Plastic and Reconstructive Surgery, Brigham and Women’s Hospital, Boston, USA

**Keywords:** climate change, disaster preparedness, environmental health, extreme weather, natural disasters, pollution, reconstructive surgery

## Abstract

The increasing frequency and severity of natural disasters, exacerbated by climate change, have significant implications for medicine, particularly in the realm of plastic and reconstructive surgery. Current research indicates that limb injuries, soft tissue trauma, and burns are among the most common injuries sustained during natural disasters. The immediate need for surgical interventions, such as wound closures, skin grafts, amputations, and limb salvage techniques, underscores the vital role of plastic and reconstructive surgery in disaster response.

We conducted a comprehensive literature search across PubMed, Embase, Global Health, and Web of Science Core Collection on March 4, 2025. All English-language original research studies that described climate-related natural disasters, extreme weather, or pollution requiring plastic and reconstructive surgery intervention were screened and included in this review. A total of 35 studies met our criteria for final analysis. A larger number of studies were published in recent years, with the majority of studies from high-income countries (n=26, 74.3%). Hand and lower extremity surgeries were the most common after a weather event (n=22, 62.9%), followed by general (n=11, 31.4%), burn (n=8, 22.9%), craniofacial (n=4, 11.4%), and breast reconstruction (n=3, 8.6%).

This scoping review highlights the growing relevance of climate-related events to the field, underscoring the need for improved emergency preparedness among plastic surgeons. As weather events continue to worsen, further research is essential to quantify their impact and guide future clinical response.

## Introduction and background

**Figure 1:**
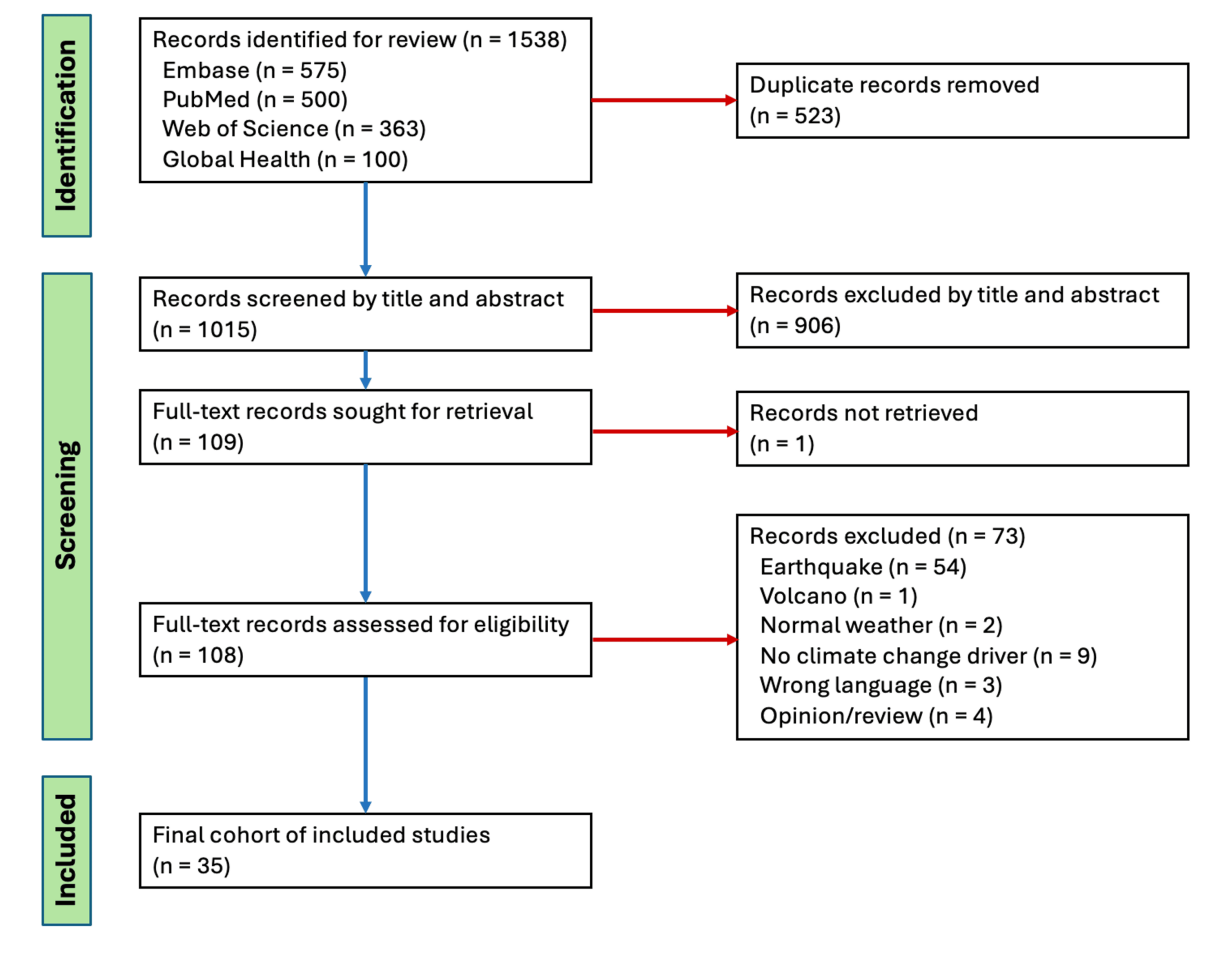
PRISMA Flow Diagram of the Scoping Review Process. PRISMA: Preferred Reporting Items for Systematic Reviews and Meta-Analyses

Climate change is a timely issue that hardly spares any part of society, and the healthcare system is no exception. In 2023, indicators of climate change revealed a consternating reality despite international attempts to counteract its unmitigated progression [1]. Compared to the pre-industrial baseline, the mean annual temperature had reached a record high, extreme precipitation had increased in 61% of the global land, and heat-related mortality of people older than 65 years increased by 167%, an unprecedented figure [1]. This intensification of climate change has been increasing the burden on the healthcare system, which has in part been attributed to the spread of infectious diseases [2,3] and the rise in chronic illnesses [4-6], mental health disorders [7], and heat-related mortality [8]. These examples are, however, by no means comprehensive as the far-reaching effects of climate change on human health remain an ongoing area of study.

A direct mechanism by which climate change results in health care needs is natural disasters. Some natural disasters, such as tropical cyclones, floods, droughts, wildfires, and polar vortexes, are intimately associated with climate change, as rising global temperatures can perturb the stability of the Earth’s atmosphere, which can give rise to such extreme weather conditions [9]. These climate-related natural disasters are capable of wreaking havoc, damaging infrastructure, inducing prolonged power outages, and causing victims injuries and trauma [10], leaving in their wake dire medical needs that must be addressed with a prompt and concerted response led at least partly by the healthcare system. Consistent with the potential devastation, prior studies have identified that climate-related natural disasters are followed by a surge in the use of medical care [11], including surgical interventions [12].

A significant fraction of the aforementioned surgical needs may fall under the scope of plastic and reconstructive surgery. The injuries that arise may require reconstruction and persistent surgical wound management [13]. As climate-related natural disasters are projected to become increasingly far-reaching in the foreseeable future [1], their relationship with the demand for plastic and reconstructive surgeries may be an important area of investigation that further defines surgeons’ stakes in climate change, informs the kinds of surgical needs that may become more commonplace, and contributes to the planning of more comprehensive disaster response. 

Thus, our scoping review attempts to achieve the following objectives: (i) provide a map of the current literature on the need for plastic and reconstructive surgeries following climate-related natural disasters, (ii) underscore specific mechanisms of injury requiring such surgical interventions that are becoming more prominent due to climate-related natural disasters, (iii) describe the complexities of socioeconomic and geographic variables at play, and (iv) identify the gaps in the literature.

## Review

Methods

Search Strategy 

We registered our search protocol in Open Science Framework (OSF registration number: 10.17605/ OSF.IO/RP9HE) prior to starting this review. In addition to following the methods for scoping reviews described by Peters et al. in 2020 [14], our search strategy adhered to the Preferred Reporting Items for Systematic Reviews and Meta-Analyses (PRISMA)-ScR reporting guidelines [15]. Studies were identified by searching PubMed (National Center for Biotechnology Information), Embase (Elsevier, embase.com), Global Health (C.A.B. International, EBSCOhost), and Web of Science Core Collection (Clarivate) on March 4, 2025. Controlled vocabulary terms (i.e., MeSH, Emtree, and CABI Thesaurus) were included when available and appropriate. The search strategies were designed and carried out by a health sciences librarian (CM). The exact search terms used in each of the databases are provided in the supplementary document (Appendix 1).

Inclusion Criteria

All original research articles discussing plastic surgical procedures associated with climate-related natural disasters and extreme weather events were included. Only English articles were included. No publication date limits were applied.

Exclusion Criteria

Studies that lacked primary data, did not include a clear climate-related weather or pollution event, and were published outside our authors’ native fluency were excluded. Studies that focused exclusively on earthquakes, tsunamis, and volcanoes were also excluded due to a lack of a well-established link with climate change.

Study Selection and Data Extraction

All articles were uploaded to Covidence systematic review software (Veritas Health Innovation Ltd., Melbourne, Australia); duplicate records were removed during the import process. Two independent reviewers screened each title and abstract to identify studies for full-text review. Of the articles that passed the abstract and title screening, we carefully reviewed the full text of each article to identify relevant studies for inclusion in our analysis. Conflicts between reviewers were resolved by discussion in all stages of screening. With our final cohort of included studies, we extracted data using pre-specified parameters including study type, weather event, date of weather event, type of injury, type of surgical intervention, total number of patients, patient age, patient sex, climate-related study findings, economic implications, duration of study period, country, and U.S. state when applicable. The full data extraction table is included in Appendix 2.

Limitations

This scoping review was conducted in accordance with the PRISMA-ScR guidelines to map the breadth of the existing evidence, rather than to evaluate the effectiveness of specific interventions or to generate quantitative summary estimates. In line with current methodological guidance for scoping reviews, we did not conduct a formal risk-of-bias or methodological quality assessment of the included studies. 

Data Analysis

We generated descriptive statistics and figures using Python, Excel, Word, and PowerPoint. Given the nature of a scoping review, we did not conduct any further meta-analyses.

Ethics Approval

Institutional Review Board approval and oversight were not required for our study as we did not work with any human subjects as part of this research.

Results

Study Characteristics

A total of 1,538 records were identified by the literature searches. One thousand fifteen unique records were available for screening after removal of duplicates. We excluded 906 articles during the title and abstract screening and an additional 73 articles following the full-text screening. In total, we included 35 studies in our final analysis (Figure 1).

Relevant study characteristics and findings of our included studies (n=35) are summarized in Table 1. The most commonly included studies in our analysis were case reports and case series (n=17, 48.6%). The next most common study types were retrospective cohort studies (n=9, 25.7%), retrospective observational studies (n=4, 11.4%), and cross-sectional studies (n=3, 8.6%).

**Table 1:** Summary of Included Studies

Study	Weather Event	Date of the Weather Event	Type of Injury	Type of Surgical Intervention	Total Number of Patients (n)	Patient Age as Reported	Patient Sex	Climate-Related Study Findings	Study Type	Duration of the Study Period
Krakauer et al. [16]	Air pollution	2016 - 2020	Non-syndromic cleft lip and/or palate	Cleft lip and palate repair	1,309	-	-	SO₂ and PM exposure were linked to increased rates of non-syndromic cleft lip/palate, highlighting air pollution as a prenatal risk factor.	Retrospective cohort	2016 - 2020
Harris et al. [17]	Air pollution	1/1/1993-11/27/2017	Increased breast cancer risk	Breast cancer reconstruction	205	48.7 (mean), 13.3 (SD)	0 M 205 F	Chronic exposure to air pollution and particulate matter mediates inflammatory processes that increase the risk of breast cancer.	Cross-sectional	1/1/1993-11/27/2017
Kim et al. [18]	Air pollution	2011 - 2019	Diabetic foot injury	Lower limb artery operations, amputation	347,543	61.69 ± 11.53 (mean)	164,593 M 182,950 F	Urban regions of South Korea exposed to greater air pollution had significantly increased risks of diabetic foot complications.	Retrospective cohort	2011 - 2019
Choudhury et al. [19]	Water pollution	1/2004 - 12/2015	Skin cancer	Resection	960	18-95	528 M 432 F	Mucocutaneous skin lesions were significantly more prevalent in populations exposed to water pollution.	Case control	1/2004 - 12/2015
Taylor et al. [20]	Wildfire	2/7/1967	Burns	Surgical excision, debridement, and skin grafting	35	10 to 70	26 M 9 F	Hospital overload post-disaster led to high patient mortality, highlighting the need for better emergency capacity and support for smaller hospitals.	Case series	-
Zeman et al. [21]	Wildfire	1/1/1988	Burns	Amputation and split skin grafts	1	51	1 M 0 F	Prosthetic/orthotic techniques can lead to excellent functional results as an alternative to surgery.	Case report	-
Cleland et al. [22]	Wildfire	2/2009	Burns	Skin grafts and escharotomy	19	31-77	14 M 5 F	Seventeen out of the 19 patients admitted following the first 48 hours of the bushfires underwent surgery, resulting in 4355 minutes of OR time in the first week.	Retrospective cohort	2/2009
Whiting et al. [23]	Extreme heat	-	Burns	Surgical excision, debridement, and skin grafting	2	66, 58	1 M 1 F	Contact pavement burns are a new mechanism of injury from climate change and result in longer hospital stays and more intensive surgical intervention compared to traditional burns.	Case series	-
Nakamura et al. [24]	Extreme heat	6/2011 - 7/2011	Dry necrosis	Debridement, skin graft, amputation, and flap	2	81, 77	1 M 1 F	Heat stress related to heatwaves may predispose patients to necrotic damage that requires surgical interventions.	Case series	6/2011 - 7/2011
Xiang et al. [25]	Extreme heat	7/01/2001 - 6/30/2010	Soft wound injuries, amputations, MSK and connective tissue diseases	-	125,267	Large Range, with many (~1/2) patients between 35-54	85138 M 40129 F	Heatwaves increased occupational burns, wounds, lacerations, and amputations, especially among older male workers.	Retrospective cohort	7/01/2001 - 6/30/2010
Lorentzen et al. [26]	Winter storm	12/2016 - 2/2017	Frostbite	Wound debridement, drainage, and amputation	6	46, 31, 44, 32, 53, 57	5M 1F	Populations in the Arctic Circle are particularly at risk of frostbite due to frequent cold temperature extremes.	Case series	12/2016 - 2/2017
Cindass et al. [27]	Winter storm	2/12/21 - 4/1/21	Frostbite	Amputation	13	35-66	13 M 0 F	Homelessness is a serious risk factor for weather exposures. Out of 13 patients, 10 considered themselves homeless; 7 out of the 13 patients required operative management.	Retrospective cohort	2/12/21 - 4/1/21
Ahmad et al. [28]	Extreme cold	-	Frostbite	Wound debridement, skin grafts, and amputation	1	23	1 M 0 F	Case of an intoxicated man who developed frostbite after walking without shoes in freezing weather.	Case report	-
Boles et al. [29]	Extreme cold	1/2007 - 4/2017	Frostbite	Skin graft, escharotomy, and amputation	47	15 (medium)	24 M 23 F	Frostbite in children was tied to lack of supervision and alcohol use, with risks rising at temperatures below −6°C.	Retrospective cohort	1/2007 - 4/2017
Roche-Nagle et al. [30]	Extreme cold	-	Frostbite, hemorrhagic blistering, and sepsis	Bilateral below-knee amputations	1	47	1 M 0 F	Frostbite case report emphasized varied causes of injury (social, occupational, recreational) and importance of clinical management knowledge.	Case report	-
Xiao et al. [31]	Extreme cold	1/2009 - 1/2019	Frostbite	Amputation	27	14 to 81	23 M 4 F	Pilgrimage is an essential and sacred ritual for Tibetan Buddhists. Nonstop travel without proper shelter risks exposures to extreme weather patterns such as blizzards.	Retrospective cohort	1/2009 - 1/2019
Xiao et al. [32]	Extreme cold	12/20/2018	Frostbite with mummified gangrene	Bilateral amputation	1	18	0 M 1 F	Case of an 18-year-old exposed to a blizzard during a pilgrimage, developed cold mummified gangrene that resulted in amputation. Treatment complicated by language barriers, ethnic tensions, and lack of education on frostbite.	Case report	-
Endorf et al. [33]	Extreme cold	2016-2018	Frostbite	Amputation	7560	51.5	6040 M 1520 F	Black race, homelessness, and substance use were linked to increased frostbite incidence and higher amputation rates.	Retrospective database analysis	2016-2018
Zhang et al. [34]	Extreme cold	10/1986 - 10/1991	Injuries from electric saw, cutting, punching, avulsion, pounding, and crush resulting	Multiple digit replantation, blood vessel and nerve repair, and amputation	130	2 to 58	102 M 28 F	Multiple-digit replantation had positive outcomes (94% survival rate). Conditions permitting, all fingers should be replanted. Warming of the frozen digit is recommended prior to debridement and replantation.	Case series	10/1986 - 10/1991
Buckle et al. [35]	Hurricane	8/25/2017 - 9/22/2017	Open fracture with soft tissue damage	Wound debridement (followed by multiple plastics procedures due to resistant infection): tissue expansion & wound closure, full thickness rotational flap, split thickness skin graft	1	25	1 M 0 F	Water-exposed open fractures in disaster settings require intensive debridement and infection prevention. Primary wound closure can lead to high rates of serious infections.	Case report	8/25/2017 - 9/22/2017
Mellgard et al. [36]	Hurricane	2017	Stump ulceration with distal necrotic tissue	-	1	59	1 M 0 F	Natural disasters do not fall within state boundaries or territory lines, but Medicare coverage can vary substantially. This patient's case from Puerto Rico illustrates the difficulty to assure continuity of care after Hurricane Maria.	Case report	2017
Muñoz-Torres et al. [37]	Hurricane	12/2019 - 3/2021	Increased breast cancer risk	Breast cancer reconstruction	241	62.6 (mean)	19 M 80 F	Cancer care delays due to natural disasters were worsened by regional disparities in Puerto Rico.	Cross sectional	12/2019 to 3/2021
Langdon et al. [38]	Landslide	1/2018	Skin and soft tissue injury, craniofacial trauma, corneal abrasion/eye irritation, orthopedic injury, pelvic fracture, spinal fracture, scapula fracture, mud impaction, burns, traumatic brain injury, wound infection, acute kidney injury	Soft tissue irrigation and debridement, wound vacuum therapy, skin graft, internal fixation of fractures, ligament/tendon repair, and body orifice irrigation	24	52.5 (median)	10 F 13 M	Debris flow syndrome is a pattern of injuries including soft tissue injuries, hypothermia, craniofacial trauma, corneal abrasions, orthopedic injuries, and mud impaction that occur after a debris flow.	Retrospective cohort	1/2018
Arango-Granados et al. [39]	Landslide	-	Crush injury	Fasciotomy, above knee amputation	1	29	1 M 0 F	Case of a patient under a landslide for 50 hours who benefited from early amputation due to progressive deterioration.	Case report	-
Carvalho et al. [40]	Landslide	-	Fractures, crush injury and soft tissue injury	Fracture fixation, soft tissue reconstruction, and amputation	11	1 in 20s-30s, 2 in 30s-40s, 4 in 40s-50s, 2 in 50-60s, 2 in 60s-70s	3 M 8 F	Rehabilitation after landslides was underused due to competing needs, limited access, and knowledge of such resources.	Cross sectional	-
Austin et al. [41]	Tornado	-	Burns and soft tissue injury	Wound debridement and skin grafting	4	50, 48, 17	2 M 2 F	Fungal burn infections have increased 10x since 1960s; survival improves with combined surgical and antifungal treatment.	Case series	-
Hartmann et al. [42]	Tornado	4/27/2011-4/28/2011	Soft tissue injury, orthopedic injury, brain hemorrhage, and abdominal and thoracic injury	Laparotomy, craniotomy, soft tissue reconstruction, orthopedic fixation, thoracostomy, thoracotomy, split-thickness skin graft, BKA, splenic embolization, dilation and curettage	28	21-87	16 M 12 F	Following a tornado, the main surgical interventions performed included soft tissue debridement and reconstruction, as well as orthopedic fixation.	Retrospective cohort	4/27/2011-4/28/2011
May et al. [43]	Tornado	5/1999	Soft tissue injury, soft tissue infection and contamination, head injuries (scalp lacerations and contusions, concussions, skull fracture, intracranial bleeding), fractures, dislocations, blunt abdominal and chest trauma, and penetrating trauma	Wound debridement, soft tissue reconstruction, fracture fixation, and neurosurgery	147	35.7 (estimated mean based on a subset of patients whose ages were recorded)	67 M 79 F 1 unspecified	This single-site experience of care for tornado-related injuries suggests complex soft tissue wounds, head injuries, and fractures are most common.	Retrospective review	5/1999
Chern et al. [44]	Tornado	4/27/11 - 4/28/11	Cranial, spine, and peripheral nerve injuries	Craniectomy, EVD placement, bedside scalp closure	24	1 month - 14 years	-	Within the first 24 hours post-tornado, 24 pediatric patients received neurosurgical care with 15 undergoing surgery.	Retrospective Cohort	4/27/11 - 4/28/11
Ozaki et al. [45]	Typhoon	2019	Skin infection	Would care (surgical drainage tube)	1	80	0 M 1 F	Case of an 80-year old post operative breast cancer patient highlights how risks including cancer diagnosis, post-operative status, and advanced age increase the likelihood of skin infection following immersion in flood waters.	Cross-sectional	2019
Kaneda et al. [46]	Typhoon	4/2019 - 9/2021	Increased breast cancer risk	Mastectomy and axillary dissection, and breast reconstruction	1	50s	0 M 1 F	Case of a Japanese woman showing signs of breast cancer; however, intervention was delayed due to Typhoon Hagibis and the COVID-19 pandemic.	Retrospective cohort	4/2019 - 9/2021
Selvaggi et al. [47]	Ball lightning	-	Burns	Surgical debridement and coverage with a split-thickness skin graft	2	28, 5	1 M 1 F	Ball lightning, a mixture of fire and electricity, caused fire and electrical burns in a father and daughter. Both recovered following medical and surgical burn treatment within 43 days.	Case control	-
Laohawiriyakamol et al. [48]	Snake bite	1/1999 - 12/2008	Soft tissue injury and compartment syndrome	Serial wound debridement, skin grafting, fasciotomy due to compartment syndrome, cutaneous resurfacing, toe amputation due to necrosis	58	2-3 yrs	43 M 15 F	Snake bites are common in children in developing countries. The majority occurred in the summer and rainy seasons (Feb-August), especially during floods.	Case series	1/1999 - 12/2008
Lee et al. [49]	Unspecified	2000 - 2011	Crushing, fracture, and internal organ rupture	-	642	51.56 (mean)	400 M 242 F	Deaths from natural disastersdisproportionately affected agricultural workers.	Case report	2000 - 2011
Naidu et al. [50]	Unspecified	1/1/2008 - 12/31/2017	Limb injury	Limb amputation	936	27 (median)	706 M 230 F	Humanitarian crises are associated with increased rates of limb amputations.	Retrospective cohort	1/1/2008 - 12/31/2017

Temporal and Geographic Distribution of Studies

**Figure 2:**
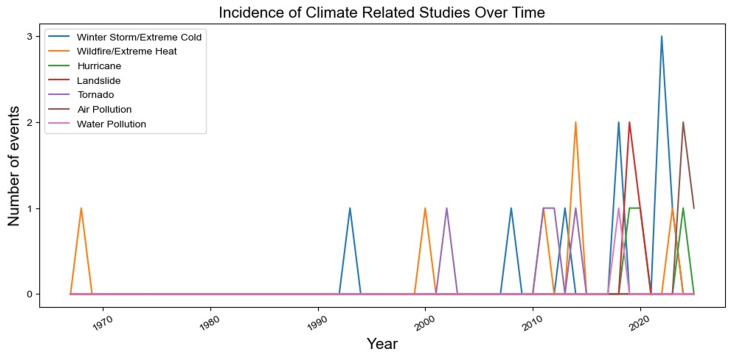
Incidence of Climate-Related Studies Over Time The figure displays the number of studies meeting our inclusion criteria published each year per climate event.

There was an increase in climate-related studies over time, with the majority of studies (n= 20, 57.1%) being published between 2015-2020 alone (Figure 2). Referencing the World Bank’s income group classification system, we found that the majority of studies were published in high-income countries (HICs) (n=26, 74.3%), followed by upper-middle-income countries (UMIC) (n=6, 17.1%), lower-middle-income countries (LMICs) (n=2, 5.7%), and low-income countries (LICs) (n=1, 2.9%) (Appendix 3). Within the U.S., we also categorized studies by the individual states and territories impacted by natural disasters, extreme weather, or pollution. Texas (n=4, 11.4%), California (n=3, 8.6%), and New York (n=3, 8.6%) had the highest number of published studies describing a climate event in the region (Appendix 2). There was one study that included all 50 U.S. states in a retrospective database analysis of extreme cold (Appendix 2).

Incidence of Climate Events Reported

The included studies reported on a range of climate-related natural disasters and extreme weather events, including hurricanes (n=3, 8.6%), tornados (n=4, 11.4%), landslides (n=3, 8.6%), typhoons (n=2, 5.7%), wildfires (n=3, 8.6%), air pollution (n=3, 8.6%), winter storms (n=2, 5.7%), extreme heat (n = 3, 8.6%), and extreme cold (n = 7, 20.0%) (Table 1). Each climate event type was associated with various plastic surgery indications and interventions, as outlined in Table 2.

**Table 2:** Plastic Surgery Indications and Interventions by Climate Event

Plastic Surgery Specialty	Percentage of Studies Inclusive of Each Specialty	Climate Event	Plastic Surgery-Specific Indication	Plastic Surgery-Specific Intervention
General Reconstruction	31.4%	Extreme heat	Dry necrosis	Flap, wound debridement, skin grafting [24]
		Winter storm	Frostbite	Wound debridement and drainage [26]
		Landslide	Soft tissue injury	Soft tissue reconstruction [40]
			Skin & soft tissue injury and infection	Wound irrigation & debridement, wound vacuum therapy, skin grafting [38]
			Crush injury	Fasciotomy [39]
		Tornado	Soft tissue injury	Soft tissue reconstruction [42,43]
				Skin grafting [42]
			Soft tissue infection	Wound debridement [43]
		Hurricane	Stump ulceration with necrotic tissue [8]	N/A
		Typhoon	Skin infection	Surgical wound care & drainage [45]
		Snake bite	Soft tissue injury, compartment syndrome	Wound debridement, skin grafting, fasciotomy, cutaneous resurfacing [48]
		Water pollution	Skin cancer	Surgical resection [19]
Hand & Lower Extremity	62.9%	Air pollution	Diabetic foot injury	Lower limb amputation [18]
		Wildfire	Burns	Amputation [21]
		Extreme heat	Dry necrosis	Amputation [24]
			Musculoskeletal and soft tissue injury	Amputation [25]
		Winter storm	Frostbite	Amputation [26,27]
		Extreme cold	Frostbite	Amputation [28,29,31-33]
			Frostbite, hemorrhagic blistering, sepsis	Amputation [30]
			Blunt trauma, crush, avulsion injuries	Amputation, multiple digit replantation, blood vessel & nerve repair [34]
		Hurricane	Open fracture	Wound debridement, tissue expansion & wound closure, flap, skin grafting [35]
		Landslide	Crush injury	Amputation [39,40]
			Fractures	Fracture fixation [38,40]
			Tendon/Ligament damage	Tendon/Ligament repair [38]
		Tornado	Fractures	Fracture fixation [42,43]
			Musculoskeletal and soft tissue injury	Amputation [42]
		Snakebite	Soft tissue injury	Toe amputation [48]
		Mixed climate events	Limb injury	Limb amputation [50]
			Crushing, fractures	N/A [49]
Craniofacial	11.4%	Air pollution	Non-syndromic cleft lip and palate	Cleft lip and palate repair [16]
		Landslide	Craniofacial trauma	Internal fracture fixation, facial reconstruction [38]
		Tornado	Head and scalp injuries	Facial reconstruction [43]
				Scalp closure [44]
Burn	22.9%	Wildfire	Burns	Skin grafting [20-22]
				Wound debridement, surgical excision [20]
				Escharotomy [22]
		Extreme heat	Burns	Wound debridement, skin grafting, and surgical excision [23]
		Ball lightning	Burns	Wound debridement and skin grafting [47]
		Tornado	Burns	Wound debridement and skin grafting [41]
		Extreme cold	Frostbite	Escharotomy [29]
				Wound debridement [28]
				Skin grafting [28,29]
Breast Reconstruction	8.6%	Air pollution	Increased breast cancer risk	Breast reconstruction [17]
		Hurricane	Increased breast cancer risk	Breast reconstruction [37]
		Typhoon	Increased breast cancer risk	Breast reconstruction [46]

Plastic and Reconstructive Surgery Interventions

When summarizing the studies, if a paper reported on interventions that applied to more than one plastic surgery subspecialty, we counted that study separately in each relevant category. Among plastic surgery subspecialties, hand and lower extremity surgery was most indicated after an acute weather event (n=22, 62.9%), with common procedures including amputation, wound closure, digit replantation, and nerve repair (Table 2). General reconstruction was also highly indicated (n=11, 31.4%) in our study cohort, as soft tissue reconstruction, skin grafts and flaps, and wound debridement were frequently performed (Table 2). Burn care (n=8, 22.9%) following wildfires, extreme heat, and lightning strikes required interventions such as wound debridement, skin graft, and escharotomy (Table 2). Additional plastic surgery indications included craniofacial (n=4, 11.4%) and breast reconstruction (n=3, 8.6%) (Table 2).

Discussion

Although disaster medicine is an entire specialty of its own, this review elucidates the lesser-described impact of climate events on plastic and reconstructive surgery to raise awareness of disaster preparedness in this field. As these events will only become more severe and commonplace in the future - and as population centers located in susceptible areas are increasingly affected - policy actions and infrastructure reinforcements should also be enacted to mitigate these effects. Our study also highlights the geographic, economic, and social factors that exacerbate the burdens of both injury and surgical care.

Geographic Disparities 

**Figure 3:**
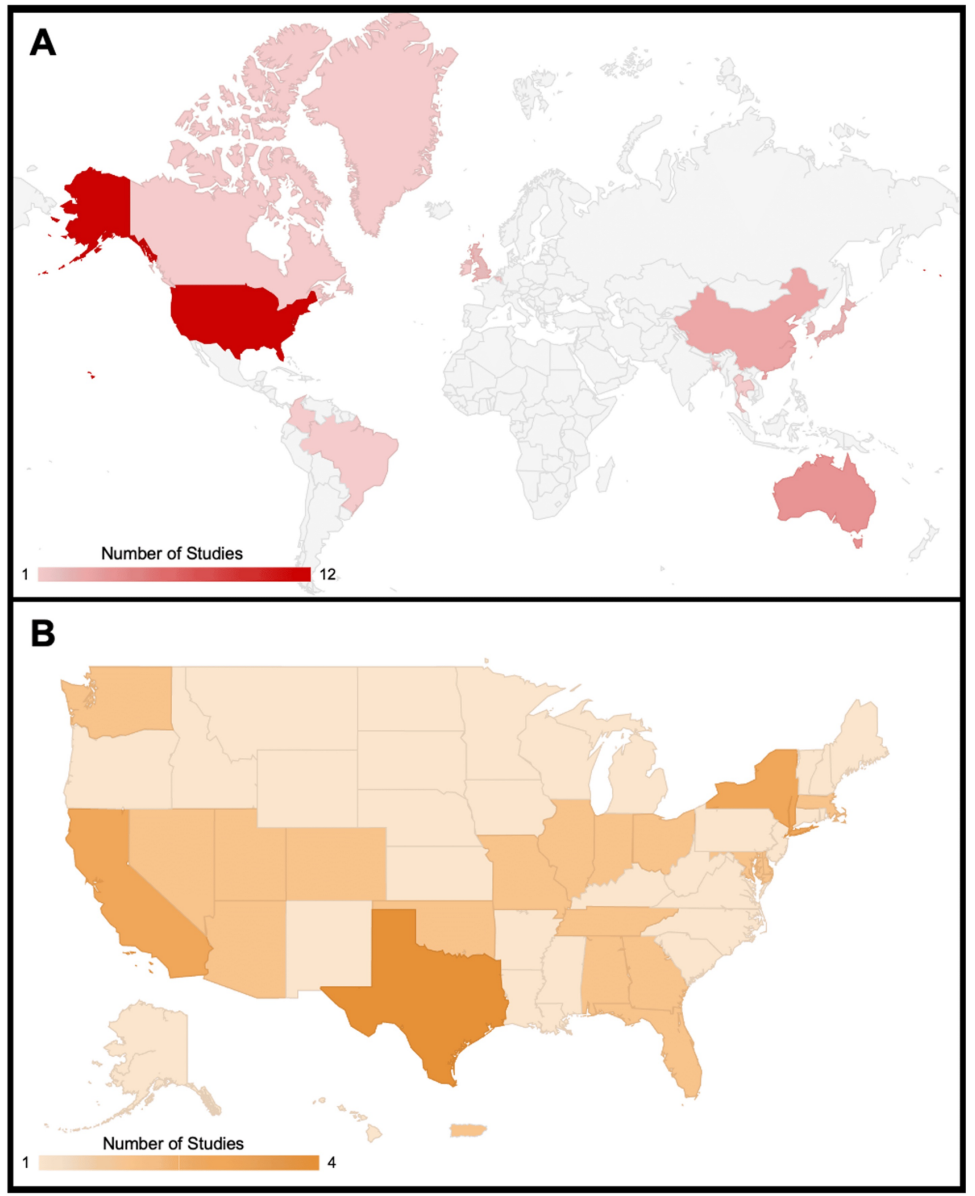
Geographic Distribution of Included Studies (A) World Map showing geographic distribution of climate events in the included studies. Countries shaded in gray had no climate event studies that fit our inclusion criteria. (B) U.S. State Map showing geographic distribution of climate events in the United States present in the included studies. The baseline level of n=1 represents a national U.S. study.

Despite the large majority of studies being published in HICs (n=26, 74.3%), with the United States alone comprising over a third of the included studies (n=12, 34.3%), the impact of natural disasters is felt throughout the world and disproportionately affects under-resourced nations, due to both their pre-event vulnerability and post-event response [51]. According to a report by the United Nations Office for Disaster Risk Reduction, 90.7% of deaths caused by natural disasters between 1996 and 2015 occurred in low- and middle-income countries [52]. Studies focusing on these countries (UMIC, LMIC, LIC) only comprised 25.7% of the included literature on this topic, which may demonstrate not only their underlying vulnerability to climate events, but also the lack of resources and literature to bolster disaster response in these areas (Figure 3a).

Within the United States, state-level trends were also observed (Figure 3b). Texas had the highest number of included studies (n=4), which examined air pollution, winter storms, extreme cold, and hurricanes. Notably, there were more studies on Texas (n=4) than all low-income and lower middle-income countries combined (n=3). States in the Midwest and Mountain West had the fewest included studies, despite being notably affected by a range of climate events such as wildfires, severe storms, air pollution, and extreme temperatures.

Socioeconomic Considerations

Lower-income individuals are generally most affected by climate events. For instance, frostbite is exacerbated by inadequate shelter, prolonged cold exposure, and community infrastructure. A single-center analysis in San Antonio, Texas examined 13 patients with frostbite impacted by the 2021 Winter Storm Uri, of which 10 (76.9%) were experiencing homelessness. This already-vulnerable population also had limited experience with the extreme cold, which may have led to their 3-day median delay in seeking treatment [27]. In a 2017 case report by Mellgard et al., a Puerto Rican patient who had developed stump ulceration with distal necrotic tissue experienced delayed care after Hurricane Maria due to managed Medicare coverage gaps in Puerto Rico [36]. Natural disasters can thereby compromise the continuity of care despite federal insurance programs and exacerbate existing socioeconomic disparities.

This study also identifies how climate events can create an increased cost burden on healthcare systems more broadly. A case series on extreme heat in the UK showed that pavement burns, a new mechanism of injury becoming more common with climate change, result in longer hospital stays and more intensive surgical intervention compared to similarly-sized traditional burns [23]. As such, contact pavement burns require more resources, resulting in a greater cost per surface area burned. Moreover, a retrospective cohort study in Australia showed that worker compensation claims rose 6.2% during heatwaves, given the increase in occupational burns, wounds, lacerations, and amputations that were especially prevalent among older male workers [25].

Other sociodemographic determinants of injury associated with climate events were also found. For example, of 13 Tibetan Buddhist patients examined in a retrospective cohort study in China, pilgrimage was overwhelmingly responsible for frostbite (n=9, 69.2%), as the endurance of extreme conditions was a sign of religious devotion [31].

These geographic, social, and economic factors have all been compounded by the increasing occurrence and severity of climate events. The majority of included studies (n= 20, 57%) were published between 2015-2020, likely reflecting not just the rising interest in studying the implications of climate change, but also the growing impact of natural disasters on our lives, and especially on those who are the most vulnerable (Figure 2).

Indirect Health Impacts

This review also shines light on both the direct and indirect impacts of climate events on the occurrence and management of cancer. A 2025 study in Maryland showed that chronic exposure to air pollution and particulate matter mediate inflammatory processes that may increase the risk of breast cancer [17]. Likewise, a 2018 study in Bangladesh demonstrated that populations exposed to water pollution had a significantly higher prevalence of mucocutaneous skin lesions that can lead to skin cancer [19]. Coastal regions and countries susceptible to heavy flooding, storms, and water pollution may have an increased oncological burden that may not be immediately obvious.

The delayed care and surgical intervention of patients with chronic diseases and cancer as a consequence of natural disasters is another indirect effect that cannot be understated. A 2024 cross-sectional study by Muñoz-Torres et al. found that cancer care delays in Puerto Rico were worsened due to climate events, leading to increased oncological risk and fewer resources for procedures such as breast reconstruction [37]. In addition, a 2022 study in Japan found that a woman with signs of breast cancer had time-sensitive interventions delayed due to Typhoon Hagibis and the COVID-19 pandemic, leading to distant bone metastases [46].

Importantly, this study identified associations between various climate events, socioeconomic and geographic risk factors, mechanisms of injury, and surgical interventions to promote awareness among plastic and reconstructive surgeons. Improving emergency preparedness in the coming years will be especially necessary as weather events are predicted to increase in frequency and severity.

Future Directions & Policy Implications

Our findings demonstrate a global need for more disaster-related education and disaster response. Already, plastic and reconstructive surgeons in Florida have launched the Surgeon Aftercare during Emergencies (SAfE) initiative after witnessing the devastating impact of local hurricanes that displace many patients and disrupt medical practices [53]. This SAfE initiative intends to provide post-operative care to patients during emergency situations using an accessible network of volunteer surgeons [53]. In LMIC and LIC countries that are most vulnerable to the effects of these disasters, national and global plastic surgery networks can be particularly crucial in organizing a coordinated medical response after a climate event. Training the healthcare workforce to better address the surgical demand in disaster-prone areas, as well as establishing a registry for weather and disaster related injuries, will be critical in ensuring future preparedness.

Limitations

There were a number of limitations in this study. First, our selection of only studies written in English likely led to the exclusion of several studies from low- and middle-income countries, and there were no studies from Africa that met our inclusion criteria. As such, certain climate events and surgical interventions may be over- or under-represented. In addition, eight of the studies that we excluded examined the 2004 Indian Ocean earthquake and tsunami that hit Southeast Asia, and there was no other single disaster with as many studies that reported on its health impacts. Many of the affected countries were middle income countries, such as Indonesia, Sri Lanka, and Thailand. Furthermore, while not the primary focus of our study, it is difficult to quantify the delayed health impacts of a natural disaster, although we did consider the delays in surgery for non-emergent procedures that increase the risks of cancer and chronic disease progression. Overall, these findings help characterize the impact of weather events as a result of climate change on the field of plastic surgery.

## Conclusions

Climate-related natural disasters and extreme weather are producing surges in demand for certain plastic and reconstructive surgeries, such as general reconstruction, upper and lower extremity surgeries, craniofacial surgeries, burn care, and breast reconstruction. This is partly due to increasing immediate trauma burden, followed by a delay in routine care and screenings that may, in turn, increase the need for future operations, such as breast cancer surgeries. Thus, the complex relationship between the rising threat of climate-related natural disasters and plastic and reconstructive surgery is a timely and relevant area of research that warrants further investigation.

## Appendices

Appendix 1: full search strategy

Search Strategy

Medline/PubMed (National Library of Medicine, NCBI)

500 records, March 3, 2025

("Natural Disasters"[Mesh:NoExp] OR "Avalanches"[Mesh] OR "Cyclonic Storms"[Mesh] OR "Droughts"[Mesh] OR "Floods"[Mesh] OR "Landslides"[Mesh] OR "Tidal Waves"[Mesh] OR "Tsunamis"[Mesh] OR "Tornadoes"[Mesh] OR "Wildfires"[Mesh] OR "Extreme Weather"[Mesh] OR "Climate Change"[Mesh:NoExp] OR "Global Warming"[Mesh] OR "Sea Level Rise"[Mesh] OR "Environmental Pollution"[Mesh:NoExp] OR "Air Pollution"[Mesh:NoExp] OR "Traffic-Related Pollution"[Mesh] OR "Water Pollution"[Mesh:NoExp] OR "Ozone Depletion"[Mesh] OR natural disaster*[tiab] OR avalanche*[tiab] OR cyclone*[tiab] OR hurricane*[tiab] OR typhoon*[tiab] OR storm[tiab] OR storms[tiab] OR monsoon*[tiab] OR blizzard*[tiab] OR drought*[tiab] OR flood*[tiab] OR landslide*[tiab] OR land slide*[tiab] OR mudslide*[tiab] OR mud slide*[tiab] OR rockslide*[tiab] OR rock slide*[tiab] OR tidal wave*[tiab] OR tsunami*[tiab] OR tornado*[tiab] OR wild fire*[tiab] OR wildfire*[tiab] OR wildland fire*[tiab] OR forest fire*[tiab] OR bush fire*[tiab] OR bushfire*[tiab] OR brush fire*[tiab] OR brushfire*[tiab] OR mega-fire*[tiab] OR extreme weather[tiab] OR weather extreme*[tiab] OR severe weather[tiab] OR extreme heat [tiab] OR heat extremes[tiab] OR "extremely hot"[tiab:~3] OR heat wave*[tiab] OR heatwave*[tiab] OR "increasing temperatures"[tiab:~3] OR rising temperature*[tiab] OR "temperature extremes"[tiab:~1] OR extreme cold[tiab] OR "extremely cold"[tiab:~3] OR cold wave*[tiab] OR cold spell*[tiab] OR cold snap*[tiab] OR "extreme precipitation"[tiab:~3] OR "increase precipitation"[tiab:~3] OR "increasing precipitation"[tiab:~3] OR climate change*[tiab] OR climatic change*[tiab] OR global warming[tiab] OR warming climate[tiab] OR climat* warming[tiab] OR sea level rise[tiab] OR sea level* rising[tiab] OR rising sea level*[tiab]

OR pollution[tiab] OR pollutant*[tiab] OR water contaminat*[tiab] OR contaminated water[tiab] OR power outage*[tiab] OR blackout*[tiab] OR ozone hole*[tiab] OR "ozone depletion"[tiab:~3])

AND

("Surgery, Plastic"[Mesh] OR "Plastic Surgery Procedures"[Mesh:NoExp] OR reconstructive surger*[tiab] OR reconstructive surgical[tiab] OR reconstruction surger*[tiab] OR surgical reconstruction*[tiab] OR plastic surger*[tiab] OR plastic surgical[tiab] OR plastic reconstruct*[tiab] OR "Amputation, Surgical"[Mesh:NoExp] OR amputation*[tiab] OR "Replantation"[Mesh:NoExp] OR replantation*[tiab] OR reimplantation*[tiab] OR "Limb Salvage"[Mesh] OR limb salvag*[tiab] OR limb sparing[tiab] OR "Peripheral Nerve Injuries/surgery"[Mesh] OR peripheral nerve reconstruction*[tiab] OR peripheral nerve repair*[tiab] OR "Tendon Transfer"[Mesh] OR tendon transfer*[tiab] OR "Bone Transplantation"[Mesh] OR bone graft*[tiab] OR bone transplant*[tiab] OR "Surgical Flaps"[Mesh:NoExp] OR soft tissue coverage[tiab] OR surgical flap*[tiab] OR "Free Tissue Flaps"[Mesh] OR free flap*[tiab] OR free tissue flap*[tiab] OR free tissue graft*[tiab] OR free tissue transfer*[tiab] OR (("Myocutaneous Flap"[Mesh] OR muscle flap*[tiab] OR musculocutaneous flap*[tiab] OR myocutaneous flap*[tiab]) AND ("Fractures, Open"[Mesh] OR open fracture*[tiab])) OR "Amputation Stumps/surgery"[Mesh] OR stump reconstruction*[tiab] OR "Hand Injuries/surgery"[Mesh] OR (("Hand"[Mesh] OR "Wrist"[Mesh] OR "Wrist Joint"[Mesh:NoExp] OR hand[tiab] OR hands[tiab] OR wrist*[tiab] OR metacarp*[tiab] OR carpal[tiab] OR finger*[tiab] OR thumb*[tiab]) AND ("Arthroplasty"[Mesh:NoExp] OR arthroplast*[tiab] OR joint reconstruction*[tiab] OR "Fracture Fixation"[Mesh] OR fracture fixation*[tiab] OR fracture reduction*[tiab] OR "Tendon Injuries/surgery"[Mesh:NoExp] OR tendon repair*[tiab] OR tendon reconstruction*[tiab])) OR (("Jaw Fractures"[Mesh] OR "Orbital Fractures"[Mesh] OR "Zygomatic Fractures"[Mesh] OR jaw fracture*[tiab] OR maxillomandibular fracture*[tiab] OR mandible fracture*[tiab] OR mandibular fracture*[tiab] OR face fracture*[tiab] OR facial fracture*[tiab] OR nasal bone fracture*[tiab] OR nasal fracture*[tiab] OR orbital fracture*[tiab] OR lefort* fracture*[tiab] OR le fort* fracture*[tiab] OR zygomaticomaxillary complex fracture*[tiab]) AND ("Fracture Fixation, Internal"[Mesh] OR "Open Fracture Reduction"[Mesh] OR internal fixation*[tiab] OR open reduction*[tiab])) OR "Soft Tissue Injuries/surgery"[Mesh] OR ((soft tissue injur*[tiab] OR soft tissue trauma*[tiab]) AND (surger*[tiab] OR surgical[tiab] OR reconstruction*[tiab])) OR "Skin Transplantation"[Mesh] OR skin graft*[tiab] OR skin transplant*[tiab] OR "Tissue Expansion"[Mesh:NoExp] OR tissue expansion*[tiab] OR (("Burns"[Mesh:NoExp] OR burn[tiab] OR burns[tiab]) AND ("Contracture"[Mesh:NoExp] OR contracture*[tiab]) AND release[tiab]) OR "Skin Neoplasms/surgery"[Mesh] OR ((melanoma*[tiab] OR skin cancer*[tiab] OR skin neoplasm*[tiab] OR skin tumor*[tiab] OR skin tumour*[tiab]) AND (removal[tiab] OR excision*[tiab] OR reconstruction*[tiab])) OR "Mohs Surgery"[Mesh] OR "mohs surgery"[tiab:~1] OR "moh's surgery"[tiab:~1] OR "Head and Neck Neoplasms/surgery"[Mesh:NoExp] OR "Glossectomy"[Mesh] OR "Mandibular Osteotomy"[Mesh] OR "Maxillary Neoplasms/surgery"[Mesh] OR "Laryngectomy"[Mesh] OR "Pharyngectomy"[Mesh] OR "Thyroidectomy"[Mesh] OR "Parotid Neoplasms/surgery"[Mesh] OR 

"Neck Dissection"[Mesh] OR glossectom*[tiab] OR mandibular osteotom*[tiab] OR mandibulectom*[tiab] OR maxillectom*[tiab] OR laryngectom*[tiab] OR pharyngectom*[tiab] OR thyroidectom*[tiab] OR parotidectom*[tiab] OR neck dissection*[tiab] OR "Mastectomy"[Mesh] OR mastectom*[tiab] OR "Mammaplasty"[Mesh] OR mammaplast*[tiab] OR mammoplast*[tiab] OR breast reconstruction*[tiab])

Embase (Elsevier, embase.com)

575 records, March 4, 2025

Advanced Search

Remove Embase mapping options

Sources:  Embase (1974 to present)

#1

'natural disaster'/de OR 'avalanche'/de OR 'drought'/de OR 'flooding'/de OR 'landslide'/de OR 'tsunami'/de OR 'storm surge'/de OR 'monsoon climate'/de OR 'wildfire'/exp OR 'severe weather'/exp OR 'climate change'/exp OR 'sea level rise'/de OR 'pollution'/de OR 'air pollution'/de OR 'greenhouse gas emission'/de OR 'traffic pollution'/de OR 'ozone depletion'/de OR 'water pollution'/de OR 'water contamination'/de OR ("natural disaster$" OR avalanche$ OR cyclone$ OR hurricane$ OR typhoon$ OR storm OR storms OR monsoon$ OR blizzard$ OR drought$ OR flood$ OR landslide$ OR "land slide$" OR mudslide$ OR "mud slide$" OR rockslide$ OR "rock slide$" OR "tidal wave$" OR tsunami$ OR tornado$ OR "wild fire$" OR wildfire$ OR "wildland fire$" OR "forest fire$" OR "bush fire$" OR bushfire$ OR "brush fire$" OR brushfire$ OR "mega-fire$" OR "extreme weather" OR "weather extreme$" OR "severe weather" OR "extreme heat" OR "heat extremes" OR (extremely NEAR/4 (hot OR cold)) OR "heat wave$" OR heatwave$ OR (increasing NEAR/4 temperatures) OR "rising temperature$" OR (temperature NEAR/3 extremes) OR "cold wave$" OR "cold spell$" OR "cold snap$" OR ((extreme OR increas*) NEAR/4 precipitation) OR "climate change$" OR "climatic change$" OR "global warming" OR "climat* warming" OR "warming climate" OR ((rise OR rising) NEAR/3 "sea level$") OR pollution OR pollutant$ OR (ozone NEAR/4 depletion) OR "ozone hole$" OR "water contaminat*" OR "contaminated water" OR "power outage$" OR blackout$):ab,ti,kw NOT ([conference abstract]/lim OR [conference paper]/lim)

#2

'plastic surgery'/de OR ("reconstructive surger*" OR "reconstructive surgical" OR "reconstruction surger*" OR "surgical reconstruction$" OR "plastic surger*" OR "plastic surgical" OR "plastic reconstruct*"):ab,ti,kw OR 'amputation'/de OR 'limb amputation'/exp OR 'foot amputation'/exp OR 'hand amputation'/exp OR 'digit amputation'/exp OR amputation$:ab,ti,kw OR 'reimplantation'/exp OR (replantation$ OR reimplantation$):ab,ti,kw OR 'limb salvage'/de OR ("limb salvag*" OR "limb sparing"):ab,ti,kw OR 'nerve reconstruction'/de OR 'peripheral nerve injury'/dm_su OR ("peripheral nerve reconstruction$" OR "peripheral nerve repair$"):ab,ti,kw OR 'tendon transfer'/de OR "tendon transfer$":ab,ti,kw OR 'surgical flaps'/de OR ("soft tissue coverage" OR "surgical flap$"):ab,ti,kw OR 'free tissue graft'/de OR ("free flap$" OR "free tissue flap$" OR "free tissue graft$" OR "free tissue transfer$"):ab,ti,kw OR (('muscle flap'/de OR ("muscle flap$" OR "musculocutaneous flap$" OR "myocutaneous flap$"):ab,ti,kw) AND ('open fracture'/de OR "open fracture$":ab,ti,kw)) OR 'amputation stump'/exp/dm_su OR "stump reconstruction$":ab,ti,kw OR 'hand injury'/exp/dm_su OR (('hand'/exp OR 'wrist'/exp OR 'hand fracture'/exp OR (hand OR hands OR wrist$ OR metacarp* OR carpal OR finger$ OR thumb$):ab,ti,kw) AND ('arthroplasty'/de OR 'finger arthroplasty'/exp OR 'hand reconstruction'/exp OR (arthroplast* OR "joint reconstruction$"):ab,ti,kw OR 'fracture fixation'/de OR ("fracture fixation$" OR "fracture reduction$"):ab,ti,kw OR 'tendon reconstruction'/de OR ("tendon repair$" OR "tendon reconstruction$"):ab,ti,kw)) OR (('face fracture'/exp OR ("jaw fracture$" OR "maxillomandibular fracture$" OR "mandible fracture$" OR "mandibular fracture$" OR "face fracture$" OR "facial fracture$" OR "nasal bone fracture$" OR "nasal fracture$" OR "orbital fracture$" OR "lefort* fracture$" OR "le fort* fracture$" OR "zygomaticomaxillary complex fracture$"):ab,ti,kw) AND ('fracture fixation'/de OR 'open fracture reduction'/de OR ("internal fixation$" OR "open reduction$"):ab,ti,kw)) OR 'soft tissue injury'/dm_su OR (("soft tissue injur*" OR "soft tissue trauma$") AND (surger* OR surgical OR reconstruction$)):ab,ti,kw OR (('burn'/de OR burn$:ab,ti,kw) AND ('skin transplantation'/exp OR ("skin graft*" OR "skin transplant*"):ab,ti,kw)) OR (('burn scar'/de OR burn$:ab,ti,kw) AND ('tissue expansion'/de OR "tissue expansion$":ab,ti,kw)) OR 'burn contracture'/dm_su OR (burn$ AND contracture$ AND release):ab,ti,kw OR 'skin tumor'/exp/dm_su OR ((melanoma$ OR skin cancer$ OR skin neoplasm$ OR skin tumor$ OR skin tumour$) AND (removal OR excision$ OR reconstruction$)):ab,ti,kw OR 'Mohs micrographic surgery'/de OR ((mohs OR "moh s") NEAR/2 surgery):ab,ti,kw OR 'head and neck tumor'/exp/dm_su OR 'glossectomy'/exp OR 'mandibulectomy'/exp OR 'maxilla resection'/de OR 'laryngectomy'/exp OR 'pharyngectomy'/exp OR 'thyroidectomy'/exp OR 'parotidectomy'/exp OR 'neck dissection'/de OR (glossectom* OR "mandibular osteotom*" OR mandibulectom* OR maxillectom* OR laryngectom* OR pharyngectom* OR thyroidectom* OR parotidectom* OR "neck dissection*"):ab,ti,kw OR 'mastectomy'/exp OR mastectom*:ab,ti,kw OR 'breast reconstruction'/de OR (mammaplast* OR mammoplast* OR "breast reconstruction*"):ab,ti,kw NOT ([conference abstract]/lim OR [conference paper]/lim)

#2

'plastic surgery'/de OR ("reconstructive surger*" OR "reconstructive surgical" OR "reconstruction surger*" OR "surgical reconstruction$" OR "plastic surger*" OR "plastic surgical" OR "plastic reconstruct*"):ab,ti,kw OR 'amputation'/de OR 'limb amputation'/exp OR 'foot amputation'/exp OR 'hand amputation'/exp OR 'digit amputation'/exp OR amputation$:ab,ti,kw OR 'reimplantation'/exp OR (replantation$ OR reimplantation$):ab,ti,kw OR 'limb salvage'/de OR ("limb salvag*" OR "limb sparing"):ab,ti,kw OR 'nerve reconstruction'/de OR 'peripheral nerve injury'/dm_su OR ("peripheral nerve reconstruction$" OR "peripheral nerve repair$"):ab,ti,kw OR 'tendon transfer'/de OR "tendon transfer$":ab,ti,kw OR 'surgical flaps'/de OR ("soft tissue coverage" OR "surgical flap$"):ab,ti,kw OR 'free tissue graft'/de OR ("free flap$" OR "free tissue flap$" OR "free tissue graft$" OR "free tissue transfer$"):ab,ti,kw OR (('muscle flap'/de OR ("muscle flap$" OR "musculocutaneous flap$" OR "myocutaneous flap$"):ab,ti,kw) AND ('open fracture'/de OR "open fracture$":ab,ti,kw)) OR 'amputation stump'/exp/dm_su OR "stump reconstruction$":ab,ti,kw OR 'hand injury'/exp/dm_su OR (('hand'/exp OR 'wrist'/exp OR 'hand fracture'/exp OR (hand OR hands OR wrist$ OR metacarp* OR carpal OR finger$ OR thumb$):ab,ti,kw) AND ('arthroplasty'/de OR 'finger arthroplasty'/exp OR 'hand reconstruction'/exp OR (arthroplast* OR "joint reconstruction$"):ab,ti,kw OR 'fracture fixation'/de OR ("fracture fixation$" OR "fracture reduction$"):ab,ti,kw OR 'tendon reconstruction'/de OR ("tendon repair$" OR "tendon reconstruction$"):ab,ti,kw)) OR (('face fracture'/exp OR ("jaw fracture$" OR "maxillomandibular fracture$" OR "mandible fracture$" OR "mandibular fracture$" OR "face fracture$" OR "facial fracture$" OR "nasal bone fracture$" OR "nasal fracture$" OR "orbital fracture$" OR "lefort* fracture$" OR "le fort* fracture$" OR "zygomaticomaxillary complex fracture$"):ab,ti,kw) AND ('fracture fixation'/de OR 'open fracture reduction'/de OR ("internal fixation$" OR "open reduction$"):ab,ti,kw)) OR 'soft tissue injury'/dm_su OR (("soft tissue injur*" OR "soft tissue trauma$") AND (surger* OR surgical OR reconstruction$)):ab,ti,kw OR (('burn'/de OR burn$:ab,ti,kw) AND ('skin transplantation'/exp OR ("skin graft*" OR "skin transplant*"):ab,ti,kw)) OR (('burn scar'/de OR burn$:ab,ti,kw) AND ('tissue expansion'/de OR "tissue expansion$":ab,ti,kw)) OR 'burn contracture'/dm_su OR (burn$ AND contracture$ AND release):ab,ti,kw OR 'skin tumor'/exp/dm_su OR ((melanoma$ OR skin cancer$ OR skin neoplasm$ OR skin tumor$ OR skin tumour$) AND (removal OR excision$ OR reconstruction$)):ab,ti,kw OR 'Mohs micrographic surgery'/de OR ((mohs OR "moh s") NEAR/2 surgery):ab,ti,kw OR 'head and neck tumor'/exp/dm_su OR 'glossectomy'/exp OR 'mandibulectomy'/exp OR 'maxilla resection'/de OR 'laryngectomy'/exp OR 'pharyngectomy'/exp OR 'thyroidectomy'/exp OR 'parotidectomy'/exp OR 'neck dissection'/de OR (glossectom* OR "mandibular osteotom*" OR mandibulectom* OR maxillectom* OR laryngectom* OR pharyngectom* OR thyroidectom* OR parotidectom* OR "neck dissection*"):ab,ti,kw OR 'mastectomy'/exp OR mastectom*:ab,ti,kw OR 'breast reconstruction'/de OR (mammaplast* OR mammoplast* OR "breast reconstruction*"):ab,ti,kw NOT ([conference abstract]/lim OR [conference paper]/lim)

(#1 AND #2)

Global Health (C.A.B. International, EBSCOhost)

100 records March 4, 2025

Advanced Search

Search Options / Publication Type = Academic Journal

S1

DE "natural disasters" OR DE "floods" OR DE "tsunamis" OR DE "avalanches" OR DE "landslides" OR DE "hurricanes" OR DE "typhoons" OR DE "tornadoes" OR DE "storms" OR DE "drought" OR DE "wildfire"

OR TI ("natural disaster#" OR avalanche# OR cyclone# OR hurricane# OR typhoon# OR storm OR storms OR monsoon# OR blizzard# OR drought# OR flood# OR landslide# OR "land slide#" OR mudslide# OR "mud slide#" OR rockslide# OR "rock slide#" OR "tidal wave#" OR tsunami# OR tornado# OR "wild fire#" OR wildfire# OR "wildland fire#" OR "forest fire#" OR "bush fire#" OR bushfire# OR "brush fire#" OR brushfire# OR "mega-fire#" OR "extreme weather" OR "weather extreme#" OR "severe weather" OR "extreme heat" OR "heat extremes" OR (extremely N3 (hot OR cold)) OR "heat wave#" OR heatwave# OR (increasing N3 temperatures) OR "rising temperature#" OR (temperature N2 extremes) OR "cold wave#" OR "cold spell#" OR "cold snap#" OR ((extreme OR increas*) N3 precipitation)) OR AB ("natural disaster#" OR avalanche# OR cyclone# OR hurricane# OR typhoon# OR storm OR storms OR monsoon# OR blizzard# OR drought# OR flood# OR landslide# OR "land slide#" OR mudslide# OR "mud slide#" OR rockslide# OR "rock slide#" OR "tidal wave#" OR tsunami# OR tornado# OR "wild fire#" OR wildfire# OR "wildland fire#" OR "forest fire#" OR "bush fire#" OR bushfire# OR "brush fire#" OR brushfire# OR "mega-fire#" OR "extreme weather" OR "weather extreme#" OR "severe weather" OR "extreme heat" OR "heat extremes" OR (extremely N3 (hot OR cold)) OR "heat wave#" OR heatwave# OR (increasing N3 temperatures) OR "rising temperature#" OR (temperature N2 extremes) OR "cold wave#" OR "cold spell#" OR "cold snap#" OR ((extreme OR increas*) N3 precipitation)) OR DE "climate change" OR DE "global warming" OR TI ("climate change#" OR "climatic change#" OR "global warming" OR "climat* warming" OR "warming climate" OR ((rise OR rising) N3 "sea level#")) OR AB ("climate change#" OR "climatic change#" OR "global warming" OR "climat* warming" OR "warming climate" OR ((rise OR rising) N2 "sea level#")) OR DE "pollution" OR DE "air pollution" OR DE "water pollution" OR DE "ozone depletion" OR TI (pollution OR pollutant* OR (ozone N3 depletion) OR "ozone hole#" OR "water contaminat*" OR "contaminated water" OR "power outage#" OR blackout#) OR AB (pollution OR pollutant* OR (ozone N3 depletion) OR "ozone hole#" OR "water contaminat*" OR "contaminated water" OR "power outage#" OR blackout#)

S2

DE "plastic surgery" OR TI ("reconstructive surger*" OR "reconstructive surgical" OR "reconstruction surger*" OR "surgical reconstruction#" OR "plastic surger*" OR "plastic surgical" OR "plastic reconstruct*") OR AB ("reconstructive surger*" OR "reconstructive surgical" OR "reconstruction surger*" OR "surgical reconstruction#" OR "plastic surger*" OR "plastic surgical" OR "plastic reconstruct*") OR DE "amputation" OR TI amputation# OR AB amputation# OR TI (replantation# OR reimplantation# ) OR AB (replantation# OR reimplantation#) OR TI ("limb salvag*" OR "limb sparing") OR AB ("limb salvag*" OR "limb sparing") OR TI ("peripheral nerve reconstruction#" OR "peripheral nerve repair#") OR AB ("peripheral nerve reconstruction#" OR "peripheral nerve repair#") OR TI "tendon transfer#" OR AB "tendon transfer#" OR TI ("bone graft*" OR "bone transplant*") OR AB ("bone graft*" OR "bone transplant*") OR TI ("soft tissue coverage" OR "surgical flap#") OR AB ("soft tissue coverage" OR "surgical flap#") OR TI ("free flap#" OR "free tissue flap#" OR "free tissue graft#" OR "free tissue transfer#") OR AB ("free flap#" OR "free tissue flap#" OR "free tissue graft#" OR "free tissue transfer#")

OR ((TI ("muscle flap#" OR "musculocutaneous flap#" OR "myocutaneous flap#") OR AB ("muscle flap#" OR "musculocutaneous flap#" OR "myocutaneous flap#")) AND (TI "open fracture#" OR AB "open fracture#")) OR TI "stump reconstruction#" OR AB "stump reconstruction#" OR ((DE "hands" OR DE "fingers" OR DE "wrists" OR TI (hand OR hands OR wrist# OR metacarp* OR carpal OR finger# OR thumb#) OR AB (hand OR hands OR wrist# OR metacarp* OR carpal OR finger# OR thumb#)) AND (DE "arthroplasty" OR DE "fracture fixation" OR TI (arthroplast* OR "joint reconstruction#" OR "fracture fixation#" OR "fracture reduction#" OR "tendon repair#" OR "tendon reconstruction#") OR AB (arthroplast* OR "joint reconstruction#" OR "fracture fixation#" OR "fracture reduction#" OR "tendon repair#" OR "tendon reconstruction#"))) OR ((TI ("jaw fracture#" OR "maxillomandibular fracture#" OR "mandible fracture#" OR "mandibular fracture#" OR "face fracture#" OR "facial fracture#" OR "nasal bone fracture#" OR "nasal fracture#" OR "orbital fracture#" OR "lefort* fracture#" OR "le fort* fracture#" OR "zygomaticomaxillary complex fracture#") OR AB ("jaw fracture#" OR "maxillomandibular fracture#" OR "mandible fracture#" OR "mandibular fracture#" OR "face fracture#" OR "facial fracture#" OR "nasal bone fracture#" OR "nasal fracture#" OR "orbital fracture#" OR "lefort* fracture#" OR "le fort* fracture#" OR "zygomaticomaxillary complex fracture#")) AND (DE "fracture fixation" OR TI ("internal fixation#" OR "open reduction#") OR AB ("internal fixation#" OR "open reduction#"))) OR ((TI ("soft tissue injur*" OR "soft tissue trauma#") OR AB ("soft tissue injur*" OR "soft tissue trauma#")) AND (TI (surger* OR surgical OR reconstruction#) OR AB (surger* OR surgical OR reconstruction#))) OR DE "skin grafting" OR TI ("skin graft*" OR "skin transplant*") OR AB ("skin graft*" OR "skin transplant*") OR TI "tissue expansion#" OR AB "tissue expansion#" OR TI (burn# AND contracture# AND release) OR AB (burn# AND contracture# AND release) OR ((DE "skin cancer" OR DE "cutaneous melanoma" OR TI (melanoma# OR skin cancer# OR skin neoplasm# OR skin tumor# OR skin tumour#) OR AB (melanoma# OR skin cancer# OR skin neoplasm# OR skin tumor# OR skin tumour#)) AND (TI (removal OR excision# OR reconstruction#) OR AB (removal OR excision# OR reconstruction#))) OR TI ((mohs OR "moh's") W1 surgery) OR AB ((mohs OR "moh's") W1 surgery) OR DE "thyroidectomy" OR TI (glossectom* OR "mandibular osteotom*" OR mandibulectom* OR maxillectom* OR laryngectom* OR pharyngectom* OR thyroidectom* OR parotidectom* OR "neck dissection*") OR AB (glossectom* OR "mandibular osteotom*" OR mandibulectom* OR maxillectom* OR laryngectom* OR pharyngectom* OR thyroidectom* OR parotidectom* OR "neck dissection*") OR DE "mastectomy" OR TI mastectom* OR AB mastectom* OR TI (mammaplast* OR mammoplast* OR "breast reconstruction#") OR AB (mammaplast* OR mammoplast* OR "breast reconstruction#")

(S1 AND S2)

Web of Science Core Collection (Clarivate)

363 records, March 4, 2025

All Editions

Advanced Search

More options - Exact search

Refine results: exclude document types: Proceeding Papers, Book Chapters, Editorial Material, Meeting Abstracts

#1

TI=("natural disaster$" OR avalanche$ OR cyclone$ OR hurricane$ OR typhoon$ OR storm OR storms OR monsoon$ OR blizzard$ OR drought$ OR flood$ OR landslide$ OR "land slide$" OR mudslide$ OR "mud slide$" OR rockslide$ OR "rock slide$" OR "tidal wave$" OR tsunami$ OR tornado$ OR "wild fire$" OR wildfire$ OR "wildland fire$" OR "forest fire$" OR "bush fire$" OR bushfire$ OR "brush fire$" OR brushfire$ OR "mega-fire$" OR "extreme weather" OR "weather extreme$" OR "severe weather" OR "extreme heat" OR "heat extremes" OR (extremely NEAR/3 (hot OR cold)) OR "heat wave$" OR heatwave$ OR (increasing NEAR/3 temperatures) OR "rising temperature$" OR (temperature NEAR/2 extremes) OR "cold wave$" OR "cold spell$" OR "cold snap$" OR ((extreme OR increas*) NEAR/3 precipitation) OR "climate change$" OR "climatic change$" OR "global warming" OR "climat* warming" OR "warming climate" OR ((rise OR rising) NEAR/2 "sea level$") OR pollution OR pollutant$ OR (ozone NEAR/3 depletion) OR "ozone hole$" OR "water contaminat*" OR "contaminated water" OR "power outage$" OR blackout$)

#2

AB=("natural disaster$" OR avalanche$ OR cyclone$ OR hurricane$ OR typhoon$ OR storm OR storms OR monsoon$ OR blizzard$ OR drought$ OR flood$ OR landslide$ OR "land slide$" OR mudslide$ OR "mud slide$" OR rockslide$ OR "rock slide$" OR "tidal wave$" OR tsunami$ OR tornado$ OR "wild fire$" OR wildfire$ OR "wildland fire$" OR "forest fire$" OR "bush fire$" OR bushfire$ OR "brush fire$" OR brushfire$ OR "mega-fire$" OR "extreme weather" OR "weather extreme$" OR "severe weather" OR "extreme heat" OR "heat extremes" OR (extremely NEAR/3 (hot OR cold)) OR "heat wave$" OR heatwave$ OR (increasing NEAR/3 temperatures) OR "rising temperature$" OR (temperature NEAR/2 extremes) OR "cold wave$" OR "cold spell$" OR "cold snap$" OR ((extreme OR increas*) NEAR/3 precipitation) OR "climate change$" OR "climatic change$" OR "global warming" OR "climat* warming" OR "warming climate" OR ((rise OR rising) NEAR/2 "sea level$") OR pollution OR pollutant$ OR (ozone NEAR/3 depletion) OR "ozone hole$" OR "water contaminat*" OR "contaminated water" OR "power outage$" OR blackout$)

#3

AK=("natural disaster$" OR avalanche$ OR cyclone$ OR hurricane$ OR typhoon$ OR storm OR storms OR monsoon$ OR blizzard$ OR drought$ OR flood$ OR landslide$ OR "land slide$" OR mudslide$ OR "mud slide$" OR rockslide$ OR "rock slide$" OR "tidal wave$" OR tsunami$ OR tornado$ OR "wild fire$" OR wildfire$ OR "wildland fire$" OR "forest fire$" OR "bush fire$" OR bushfire$ OR "brush fire$" OR brushfire$ OR "mega-fire$" OR "extreme weather" OR "weather extreme$" OR "severe weather" OR "extreme heat" OR "heat extremes" OR (extremely NEAR/3 (hot OR cold)) OR "heat wave$" OR heatwave$ OR (increasing NEAR/3 temperatures) OR "rising temperature$" OR (temperature NEAR/2 extremes) OR "cold wave$" OR "cold spell$" OR "cold snap$" OR ((extreme OR increas*) NEAR/3 precipitation) OR "climate change$" OR "climatic change$" OR "global warming" OR "climat* warming" OR "warming climate" OR ((rise OR rising) NEAR/2 "sea level$") OR pollution OR pollutant$ OR (ozone NEAR/3 depletion) OR "ozone hole$" OR "water contaminat*" OR "contaminated water" OR "power outage$" OR blackout$)

#4

TI=("reconstructive surger*" OR "reconstructive surgical" OR "reconstruction surger*" OR "surgical reconstruction$" OR "plastic surger*" OR "plastic surgical" OR "plastic reconstruct*" OR amputation$ OR replantation$ OR reimplantation$ OR "limb salvag*" OR "limb sparing" OR "peripheral nerve reconstruction$" OR "peripheral nerve repair$" OR "tendon transfer$" OR "soft tissue coverage" OR "surgical flap$" OR "free flap$" OR "free tissue flap$" OR "free tissue graft$" OR "free tissue transfer$" OR (("muscle flap$" OR "musculocutaneous flap$" OR "myocutaneous flap$") AND "open fracture$") OR "stump reconstruction$" OR (((hand OR hands OR wrist$ OR metacarp* OR carpal OR finger$ OR thumb$)) AND (arthroplast* OR "joint reconstruction$" OR "fracture fixation$" OR "fracture reduction$" OR "tendon repair$" OR "tendon reconstruction$")) OR (("jaw fracture$" OR "maxillomandibular fracture$" OR "mandible fracture$" OR "mandibular fracture$" OR "face fracture$" OR "facial fracture$" OR "nasal bone fracture$" OR "nasal fracture$" OR "orbital fracture$" OR "lefort* fracture$" OR "le fort* fracture$" OR "zygomaticomaxillary complex fracture$") AND ("internal fixation$" OR "open reduction$")) OR (("soft tissue injur*" OR "soft tissue trauma$") AND (surger* OR surgical OR reconstruction$)) OR (burn$ AND ("skin graft*" OR "skin transplant*")) OR (burn$ AND "tissue expansion$") OR (burn$ AND contracture$ AND release) OR ((melanoma$ OR skin cancer$ OR skin neoplasm$ OR skin tumor$ OR skin tumour$) AND (removal OR excision$ OR reconstruction$)) OR ((mohs OR "moh s") NEAR/1 surgery) OR glossectom* OR "mandibular osteotom*" OR mandibulectom* OR maxillectom* OR laryngectom* OR pharyngectom* OR thyroidectom* OR parotidectom* OR "neck dissection*" OR mastectom* OR mammaplast* OR mammoplast* OR "breast reconstruction*")

#5 

AB=("reconstructive surger*" OR "reconstructive surgical" OR "reconstruction surger*" OR "surgical reconstruction$" OR "plastic surger*" OR "plastic surgical" OR "plastic reconstruct*" OR amputation$ OR replantation$ OR reimplantation$ OR "limb salvag*" OR "limb sparing" OR "peripheral nerve reconstruction$" OR "peripheral nerve repair$" OR "tendon transfer$" OR "soft tissue coverage" OR "surgical flap$" OR "free flap$" OR "free tissue flap$" OR "free tissue graft$" OR "free tissue transfer$" OR (("muscle flap$" OR "musculocutaneous flap$" OR "myocutaneous flap$") AND "open fracture$") OR "stump reconstruction$" OR (((hand OR hands OR wrist$ OR metacarp* OR carpal OR finger$ OR thumb$)) AND (arthroplast* OR "joint reconstruction$" OR "fracture fixation$" OR "fracture reduction$" OR "tendon repair$" OR "tendon reconstruction$")) OR (("jaw fracture$" OR "maxillomandibular fracture$" OR "mandible fracture$" OR "mandibular fracture$" OR "face fracture$" OR "facial fracture$" OR "nasal bone fracture$" OR "nasal fracture$" OR "orbital fracture$" OR "lefort* fracture$" OR "le fort* fracture$" OR "zygomaticomaxillary complex fracture$") AND ("internal fixation$" OR "open reduction$")) OR (("soft tissue injur*" OR "soft tissue trauma$") AND (surger* OR surgical OR reconstruction$)) OR (burn$ AND ("skin graft*" OR "skin transplant*")) OR (burn$ AND "tissue expansion$") OR (burn$ AND contracture$ AND release) OR ((melanoma$ OR skin cancer$ OR skin neoplasm$ OR skin tumor$ OR skin tumour$) AND (removal OR excision$ OR reconstruction$)) OR ((mohs OR "moh s") NEAR/1 surgery) OR glossectom* OR "mandibular osteotom*" OR mandibulectom* OR maxillectom* OR laryngectom* OR pharyngectom* OR thyroidectom* OR parotidectom* OR "neck dissection*" OR mastectom* OR mammaplast* OR mammoplast* OR "breast reconstruction*")

#6

AK=("reconstructive surger*" OR "reconstructive surgical" OR "reconstruction surger*" OR "surgical reconstruction$" OR "plastic surger*" OR "plastic surgical" OR "plastic reconstruct*" OR amputation$ OR replantation$ OR reimplantation$ OR "limb salvag*" OR "limb sparing" OR "peripheral nerve reconstruction$" OR "peripheral nerve repair$" OR "tendon transfer$" OR "soft tissue coverage" OR "surgical flap$" OR "free flap$" OR "free tissue flap$" OR "free tissue graft$" OR "free tissue transfer$" OR (("muscle flap$" OR "musculocutaneous flap$" OR "myocutaneous flap$") AND "open fracture$") OR "stump reconstruction$" OR (((hand OR hands OR wrist$ OR metacarp* OR carpal OR finger$ OR thumb$)) AND (arthroplast* OR "joint reconstruction$" OR "fracture fixation$" OR "fracture reduction$" OR "tendon repair$" OR "tendon reconstruction$")) OR (("jaw fracture$" OR "maxillomandibular fracture$" OR "mandible fracture$" OR "mandibular fracture$" OR "face fracture$" OR "facial fracture$" OR "nasal bone fracture$" OR "nasal fracture$" OR "orbital fracture$" OR "lefort* fracture$" OR "le fort* fracture$" OR "zygomaticomaxillary complex fracture$") AND ("internal fixation$" OR "open reduction$")) OR (("soft tissue injur*" OR "soft tissue trauma$") AND (surger* OR surgical OR reconstruction$)) OR (burn$ AND ("skin graft*" OR "skin transplant*")) OR (burn$ AND "tissue expansion$") OR (burn$ AND contracture$ AND release) OR ((melanoma$ OR skin cancer$ OR skin neoplasm$ OR skin tumor$ OR skin tumour$) AND (removal OR excision$ OR reconstruction$)) OR ((mohs OR "moh s") NEAR/1 surgery) OR glossectom* OR "mandibular osteotom*" OR mandibulectom* OR maxillectom* OR laryngectom* OR pharyngectom* OR thyroidectom* OR parotidectom* OR "neck dissection*" OR mastectom* OR mammaplast* OR mammoplast* OR "breast reconstruction*")

(#1 OR #2 OR #3) AND (#4 OR #5 OR #6)

Numbers for your flow diagram of studies selected:

Retrieval by database:

PubMed - 500

Embase - 575

Global Health - 100

Web of Science - 363

Total retrieval - 1538

Duplicates removed - 523

Total unique records for screening, title and abstract - 1015

Appendix 2: full data extraction

**Table 3:** Full Data Extraction

Study	Weather Event	Date of Weather Event	Type of Injury	Type of Surgical Intervention	Total Patients (n)	Patient Age as Reported	Patient Sex	Climate Related Study Findings	Economic Implications	Study Type	Duration of Study Period	Country	World bank Income Group	US State
Krakauer et al [16]	Air pollution	2016 - 2020	Non syndromic cleft lip and/or palate	Cleft lip and palate repair	1,309	-	-	SO₂ and PM exposure were linked to increased rates of non- syndromic cleft lip/palate, highlighting air pollution as a prenatal risk factor.	-	Retrospective Cohort	2016 - 2020	United States	HIC	TX, AZ, CA, IL, MA, NV, UT, WA, CO, NY, FL, OH, IN, AZ, DE
Harris et al. [17]	Air pollution	1/1/1993- 11/27/2017	Increased breast cancer risk	Breast cancer reconstruction	205	48.7 (mean), 13.3 (SD)	0 M 205 F	Chronic exposure to air pollution and particulate matter mediate inflammatory processes that increase the risk of breast cancer.	-	Cross- sectional	1/1/1993- 11/27/20 17	United States	HIC	MD
Kim et al. [18]	Air pollution	2011 - 2019	Diabetic foot injury	Lower limb artery operations, amputation	347,543	61.69 ± 11.53 (mean)	164,593 M 182,950 F	Urban regions of South Korea exposed to greater air pollution had significantly increased risks of diabetic foot complications.	Diabetic foot complications in Korea have had a 47% increase in healthcare costs from 2011 to 2016.	Retrospective Cohort	2011 - 2019	South Korea	HIC	-
Choudhury et al. [19]	Water pollution	1/2004 - 12/2015	Skin cancer	Resection	960	18-95	528 M 432 F	Mucocutaneous skin lesions were significantly more prevalent in populations exposed to water pollution.	-	Case Control	1/2004 - 12/2015	Bangladesh	LMIC	-
Taylor et al. [20]	Wildfire	2/7/1967	Burns	Surgical excision, debridement, and skin grafting	35	10 to 70	26 M 9 F	Hospital overload post-disaster led to high patient mortality, highlighting the need for better emergency capacity and support for smaller hospitals.	-	Case Series	-	Australia	HIC	-
Zeman et al. [21]	Wildfire	1/1/1988	Burns	Amputation, split skin grafts	1	51	1 M 0 F	Prosthetic/orthotic techniques can lead to excellent functional results as an alternative to surgery.	Prosthetic care had lower upfront costs than surgery and may offer long-term savings despite replacement needs.	Case Report	-	Australia	HIC	-
Cleland et al. [22]	Wildfire	2/2009	Burns	Skin grafts, escharotomy	19	31-77	14 M 5 F	Seventeen out of the 19 patients admitted following the first 48 hours of the bush fires underwent surgery, resulting in 4355 minutes of OR time in the first week.	-	Retrospective Cohort	2/2009	Australia	HIC	-
Whiting et al. [23]	Extreme heat	-	Burns	Surgical excision, debridement, and skin grafting	2	66, 58	1 M 1 F	Contact pavement burns are a new mechanism of injury from climate change that result in longer hospital stays and more intensive surgical intervention compared to traditional burns.	Compared to similarly sized scald or flame burns, contact pavement burns require more resources resulting in a greater cost per surface area burned.	Case Series	-	United Kingdom	HIC	-
Nakamura et al. [24]	Extreme heat	6/2011 - 7/2011	Dry necrosis	Debridement, skin graft, amputation, flap	2	81, 77	1 M 1 F	Heat stress related to heatwaves may predispose patients to necrotic damage that require surgical interventions.	-	Case Series	6/2011 – 7/2011	-	-	-
Xiang et al. [25]	Extreme heat	7/01/2001 - 6/30/2010	Soft wound injuries, amputations, MSK and connective tissue diseases	-	125,267	Large range, with many (~1/2 ) patients between 35-54	85138 M 40129 F	Heatwaves increased occupational burns, wounds, lacerations, and amputations, especially among older male workers.	Worker compensation claims rose 6.2% during heatwaves for outdoor industry workers, though overall claims remained stable.	Retrospective Cohort	7/01/2001 - 6/30/2010	Australia	HIC	-
Lorentzen et al. [26]	Winter Storm	12/2016 – 2/2017	Frostbite	Wound debridement, drainage, amputation	6	46, 31, 44, 32, 53, 57	5 M 1 F	Populations in the Arctic Circle are particularly at risk of frostbite due to frequent cold temperature extremes.	-	Case Series	12/2016-2/2017	Greenland	HIC	-
Cindass et al. [27]	Winter storm	2/12/21 - 4/1/21	Frostbite	Amputation	13	35-66	13 M 0 F	Homelessness is a serious risk factor for weather exposures. Out of 13 patients, 10 considered themselves homeless; 7 out of the 13 patients required operative management.	-	Retrospective Cohort	2/12/21 - 4/1/21	United States	HIC	TX
Ahmad et al. [28]	Extreme cold	-	Frostbite	Wound debridement, skin grafts, amputation	1	23	1 M 0 F	Case of an intoxicated man who developed frostbite after walking without shoes in freezing weather.	-	Case Report	-	United Kingdom	HIC	-
Boles et al. [29]	Extreme cold	1/2007 - 4/2017	Frostbite	Skin graft, escharotomy, amputation	47	15 (medium)	24 M 23 F	Frostbite in children was tied to lack of supervision and alcohol use, with risks rising at temperatures below −6°C.	-	Retrospective Cohort	1/2007 - 4/2017	Canada	HIC	-
Roche-Nagle et al. [30]	Extreme cold	-	Frostbite, hemorrhagic blistering, and sepsis	Bilateral below knee amputations	1	47	1 M 0 F	Frostbite case report emphasized varied causes of injury (social, occupational, recreational) and importance of clinical management knowledge.	-	Case Report	-	Ireland	HIC	-
Xiao et al. [31]	Extreme cold	1/2009 - 1/2019	Frostbite	Amputation	27	14 to 81	23 M 4 F	Pilgrimage is an essential and sacred ritual for Tibetan Buddhists. Nonstop travel without proper shelter risks exposures to extreme weather patterns such as blizzards.	-	Retrospective Cohort	1/2009 - 1/2019	China	UMIC	-
Xiao et al. [32]	Extreme cold	12/20/2018	Frostbite with mummified gangrene	Bilateral amputation	1	18	0 M 1 F	Case of an 18-year-old exposed to a blizzard during a pilgrimage, developed cold mummified gangrene that resulted in amputation. Treatment complicated by language barriers, ethnic tensions, and lack of education on frostbite.	-	Case Report	-	China	UMIC	-
Endorf et al. [33]	Extreme cold	2016-2018	Frostbite	Amputation	7560	51.5	6040 M 1520 F	Black race, homelessness, and substance use were linked to increased frostbite incidence and higher amputation rates.	-	Retrospective Database Analysis	2016-2018	United States	HIC	All
Zhang et al. [34]	Extreme cold	10/1986 - 10/1991	Injuries from electric saw, cutting, punching, avulsion, pounding, and crush resulting	Multiple digit replantation, blood vessel and nerve repair, amputation	130	2 to 58	102 M 28 F	Multiple-digit replantation had positive outcomes (94% survival rate). Conditions permitting, all fingers should be replanted. Warming of the frozen digit is recommended prior to debridement and replantation.	-	Case Series	10/1986 - 10/1991	China	UMIC	-
Buckle et al. [35]	Hurricane	8/25/2017 - 9/22/2017	Open fracture with soft tissue damage	Wound debridement (followed by multiple plastics procedures due to resistant infection): tissue expansion, wound closure, full thickness rotational flap, split thickness skin graft	1	25	1 M 0 F	Water-exposed open fractures in disaster settings require intensive debridement and infection prevention. Primary wound closure can lead to high rates of serious infections.	-	Case Report	8/25/2017 - 9/22/2017	United States	HIC	TX
Mellgard et al. [36]	Hurricane	2017	Stump ulceration with distal necrotic tissue	-	1	59	1 M 0 F	Natural disasters do not fall within state boundaries or territory lines, but managed Medicare coverage can vary substantially. This patient's case from Puerto Rico illustrates the difficulty to assure continuity of care after Hurricane Maria.	Managed Medicare coverage gaps in Puerto Rico led to delayed care post-Hurricane Maria, despite federal insurance.	Case Report	2017	United States	HIC	Puerto Rico/NY
Muñoz- Torres et al. [37]	Hurricane	12/2019 - 3/2021	Increased breast cancer risk	Breast cancer reconstruction	241	62.6 (mean)	19 M 80 F	Cancer care delays due to natural disasters were worsened by regional disparities in Puerto Rico.	-	Cross Sectional	12/2019 to 3/2021	United States	HIC	Puerto Rico
Langdon et al. [38]	Landslide	1/2018	Skin and soft tissue injury, craniofacial trauma, corneal abrasion/eye irritation, orthopedic injury, pelvic fracture, spinal fracture, scapula fracture, mud impaction, burns, traumatic brain injury, wound infection, acute kidney injury	Soft tissue irrigation and debridement, wound vacuum therapy, skin graft, internal fixation of fractures, ligament/tendon repair, body orifice irrigation	24	52.5 (median)	10 F 13 M	Debris flow syndrome is a pattern of injuries including soft tissue injuries, hypothermia, craniofacial trauma, corneal abrasions, orthopedic injuries, and mud impaction that occur after a debris flow.	-	Retrospective Cohort	1/2018	United States	HIC	CA
Arango- Granados et al. [39]	Landslide	-	Crush injury	Fasciotomy, above knee amputation	1	29	1 M 0 F	Case of a patient under a landslide for 50 hours who benefited from early amputation due to progressive deterioration.	-	Case Report	-	Colombia	UMIC	-
Carvalho et al. [40]	Landslide	-	Fractures, crush injury, soft tissue injury	Fracture fixation, soft tissue reconstruction, amputation	11	1 in 20s- 30s, 2 in 30s-40s, 4 in 40s- 50s, 2 in 50-60s, 2 in 60s- 70s	3 M 8 F	Rehabilitation after landslides was underused due to competing needs, limited access, and knowledge of such resources.	Most patients lacked private insurance, and many lost employment by 2015 due to lasting disability.	Cross Sectional	-	Brazil	UMIC	-
Austin et al. [41]	Tornado	-	Burns, soft tissue injury	Wound debridement, skin grafting	4	50, 48, 17, 75	2 M 2 F	Fungal burn infections have increased 10x since 1960s; survival improves with combined surgical and antifungal treatment.	-	Case Series	-	United States	HIC	MO
Hartmann et al. [42]	Tornado	4/27/2011-4/28/2011	Soft tissue injury, orthopedic injury, brain hemorrhage, abdominal and thoracic injury	Laparotomy, craniotomy, soft tissue reconstruction, orthopedic fixation, thoracostomy, thoracotomy, split- thickness skin graft, BKA, splenic embolization, dilation and curettage	28	21-87	16 M 12 F	Following a tornado, the main surgical interventions performed included soft tissue debridement and reconstruction, as well as orthopedic fixation.	There appeared to be a trend with uninsured patients having worse injury, but the difference was not statistically significant (p value = 0.18).	Retrospective Cohort	4/27/2011-4/28/2011	United States	HIC	GA, TN
May et al. [43]	Tornado	5/1999	Soft tissue injury, soft tissue infection and contamination, head injuries (scalp lacerations and contusions, concussions, skull fracture, intracranial bleeding), fractures, dislocations, blunt abdominal and chest trauma, and penetrating trauma	Wound debridement, soft tissue reconstruction, fracture fixation, neurosurgery	147	35.7 (estimated mean based on a subset of patients whose ages were recorded)	67 M 79 F 1 unspecified	This single-site experience of care for tornado-related injuries suggests complex soft tissue wounds, head injuries, and fractures are most common.	-	Retrospective Review	5/1999	United States	HIC	OK
Chern et al. [44]	Tornado	4/27/11-4/28/11	Cranial, spine, and peripheral nerve injuries	Craniectomy, EVD placement, bedside scalp closure	24	1 month – 14 years	-	Within the first 24 hours post-tornado, 24 pediatric patients received neurosurgical care with 15 undergoing surgery.	-	Retrospective Cohort	4/27/11 - 4/28/11	United States	HIC	AL
Ozaki et al. [45]	Typhoon	2019	Skin infection	Wound care (surgical drainage tube)	1	80	0 M 1 F	Case of an 80-year old post operative breast cancer patient highlights how risks including cancer diagnosis, post-operative status, and advanced age increase the likelihood of skin infection following immersion in flood waters.	-	Cross sectional	2019	Japan	HIC	-
Kaneda et al. [46]	Typhoon	4/2019 – 9/2021	Increased breast cancer risk	Mastectomy and axillary dissection, breast reconstruction	1	50s	0 M 1 F	Case of a Japanese woman showing signs of breast cancer; however, intervention was delayed due to Typhoon Hagibis and the COVID-19 pandemic.	-	Retrospective Cohort	4/2019 – 9/2021	Japan	HIC	-
Selvaggi et al. [47]	Ball lightning	-	Burns	Surgical debridement and coverage with a split-thickness skin graft	2	28, 5	1 M 1 F	Ball lightening, a mixture of fire and electricity, caused fire and electrical burns in a father and daughter. Both recovered following medical and surgical burn treatment within 43 days.	-	Case Control	-	Belgium	HIC	-
Laohawiriyakamol et al. [48]	Snake bite	1/1999 – 12/2008	Soft tissue injury, compartment syndrome	Serial wound debridement, skin grafting, fasciotomy due to compartment syndrome, cutaneous resurfacing, toe amputation due to necrosis	58	2-3 yrs	43 M 15 F	Snake bites are common in children in developing countries. The majority occurred in the summer and rainy seasons (Feb- August), especially during floods.	-	Case Series	1/1999 – 12/2008	Thailand	UMIC	-
Lee et al. [49]	Unspecified^1^	2000-2011	Crushing, fracture, internal organ rupture	-	642	51.56 (mean)	400 M 242 F	Deaths from natural disasters disproportionately affected agricultural workers.	Agricultural families in Vietnam suffered more physical and economic loss from weather-related disasters.	Case Report	2000-2011	South Korea	HIC	-
Naidu et al. [50]	Unspecified^2^	1/1/2008 - 12/31/2017	Limb injury	Limb amputation	936	27 (medium)	706 M 203 F	Humanitarian crises are associated with increased rates of limb amputations.	-	Retrospective Cohort	1/1/2008-12/31/2017	Low and middle income countries	LIC/LMIC	-

Appendix 3: World Bank country income classification of included studies

**Table 4:** World Bank Country Income Classification of Included Studies Countries were identified by their income level using the World Bank’s 2024-2025 country classification system of high income countries (HICs), upper middle income countries (UMICs), lower middle income countries (LMICs), and low income countries (LICs). Note: Out of the 35 total studies, one study did not report a location and is excluded from this analysis. Another study included data from both LMICs/LICs and is counted twice.

World Bank Country Income Classification	Number of Studies	Frequency of Studies
High-income countries (HICs)	26	74.3%
Upper-middle-income countries (UMICs)	6	17.1%
Lower middle income countries (LMICs)	2	5.7%
Low-income countries (LICs)	1	2.9%
Total	35	100%
